# Individualized lifestyle intervention in PCOS women (IPOS): a study protocol for a multicentric randomized controlled trial for evaluating the effectiveness of an individualized lifestyle intervention in PCOS women who wish to conceive

**DOI:** 10.1186/s13063-023-07466-y

**Published:** 2023-07-18

**Authors:** Neena Malhotra, Taruna Arora, Vanita Suri, Saubhagya Kumar Jena, Asha Verma, Mahasampath Gowri, Nitin Kapoor, Manjeet Singh Chalga, Bharati Kulkarni, Mohan S. Kamath

**Affiliations:** 1 Division of Reproductive Medicine, Department of Obstetrics and Gynaecology, All India Institute of Medical Sciences, New Delhi, India; 2 Division of Reproductive and Child Health and Nutrition, Indian Council of Medical Research, New Delhi, India; 3 Department of Obstetrics and Gynaecology, Post-Graduate Institute of Medical Education and Research, Chandigarh, India; 4 Department of Obstetrics and Gynaecology, All India Institute of Medical Sciences, Bhubaneswar, Odisha India; 5 Department of Obstetrics and Gynaecology, Sawai Man Singh Medical College, Jaipur, Rajasthan India; 6 Department of Biostatistics, Christian Medical College, Vellore, Tamil Nadu India; 7 Department of Endocrinology, Diabetes and Metabolism, Christian Medical College, Vellore, India; 8 Non-Communicable Disease Unit, Baker Heart and Diabetes Institute, Melbourne, VIC Australia; 9 Division of Biomedical Informatics, Indian Council of Medical Research, New Delhi, India; 10 Department of Reproductive Medicine and Surgery, Christian Medical College, Vellore, Tamil Nadu India

## Abstract

**Background:**

Polycystic ovary syndrome (PCOS) is a common endocrine condition which affects women in the reproductive age group. South Asian women with PCOS have a higher risk of insulin resistance and metabolic disorder compared to women from other ethnic backgrounds. Lifestyle interventions such as dietary advice and physical exercise are recommended as a first-line management option for infertile women with PCOS. Most of the randomized controlled trials evaluating the role of lifestyle interventions in infertile PCOS women were characterized by methodological issues. The uptake of lifestyle modifications as a treatment strategy in the South Asian population is complicated by a difficult-to-change conventional high-carbohydrate diet and limited availability of space for physical activity in the region.

**Methods:**

The study is designed as an open-label, multicentre, randomized controlled trial in South Asian women with PCOS. Women attending the fertility clinic will be screened for eligibility, and women aged between 19 and 37 years who have been diagnosed with PCOS and wishing to conceive will be invited to participate in the trial. We will include women with body mass index (BMI) between ≥ 23 and ≤ 35 kg/m^2^ and duration of infertility ≤ 3 years. We plan to randomize women with PCOS into two groups: group A will receive the intervention which will consist of individualized advice on diet and physical exercise along with a telephonic reminder system and follow-up visits, and group B (control) will receive one-time advice on diet and physical exercise. Both groups will receive up to three cycles of ovulation induction with letrozole after 3 months of randomization during the 6-month treatment period. The primary outcome of the trial will be the live birth following conception during the intervention period. The secondary outcomes include clinical pregnancy rate, ongoing pregnancy rate, miscarriage rate, ectopic pregnancy rate, stillbirth, time to pregnancy, mean weight loss, differences in anthropometric parameters, improvement in menstrual regularity and quality of life score.

**Discussion:**

The IPOS trial results could help clarify and provide more robust evidence for advocating an individualized lifestyle intervention in PCOS women who wish to conceive.

**Trial registration:**

Clinical Trial Registry of India CTRI/2023/04/051620. Registered on 13 April 2023.

**Supplementary Information:**

The online version contains supplementary material available at 10.1186/s13063-023-07466-y.

## Background

Polycystic ovary syndrome (PCOS) is a common endocrine disorder which affects women in the reproductive age group [1]. Worldwide, 1.55 million new cases of PCOS among women aged between 15 and 49 years were reported in 2017 which translated into an increase of 4.47% within a time period of 10 years [2]. The incidence of PCOS was 82.44 per 100,000 in 1 year (2017) among women in the reproductive age group [2]. There are geographical differences in the prevalence of PCOS [2]. Differences in the prevalence due to ethnicity also have been observed with a higher prevalence of PCOS observed among women from South Asian ethnicity residing in the UK compared with Caucasian women [3]. Furthermore, South Asian women with PCOS have a higher risk of insulin resistance and metabolic disorder compared to women from other ethnic backgrounds [3, 4]. These variations in PCOS prevalence due to ethnicity and geographical location have been attributed to obesity, genetic predisposition, phenotypic differences, health care access and awareness [2–4].

The underlying insulin resistance and the resultant hyperinsulinaemia are considered one of the important pathophysiological factors for PCOS [5]. Hyperinsulineamia is linked to an increase in the production of androgen and lower sex hormone-binding globulin (SHBG), resulting in excess free androgens [5]. The increased level of free androgen contributes to most of the clinical features of PCOS, such as hirsutism, acne and ovulatory dysfunction [6, 7]. More specifically, the increased levels of androgen and luteinizing hormone (LH) hypersecretion at the ovarian level leads to anovulation and oligo-ovulation, which clinically manifests as infertility in these women with PCOS [8]. Overweight and obesity worsen the underlying metabolic disturbance and clinical symptoms of PCOS [9].

Lifestyle interventions such as dietary advice and physical exercise are recommended as a first-line management option for infertile women with PCOS [6, 9]. There is a strong rationale for encouraging weight loss following lifestyle modifications as it helps improve menstrual symptoms as well as increase chances of conception in women with PCOS [10].

Several trials have been conducted in the European, North American and Australian populations evaluating the efficacy of lifestyle interventions in PCOS women [11–15]. In an open-label three-arm, randomized controlled trial (RCT) conducted at two sites in the USA (*n* = 149), the investigators compared the effectiveness of preconception lifestyle intervention before ovulation induction in PCOS women [11]. The lifestyle intervention included calorie-restricted meals, medication for weight loss and physical exercise, and the treatment period was 16 weeks. The study reported significant weight loss following lifestyle intervention and higher ovulation rates. In another single-centre RCT (*n* = 183) from The Netherlands, the authors compared lifestyle intervention in the form of diet, exercise and cognitive behavioural therapy with or without telephonic reminders versus the usual care for a period of 12 months in PCOS women who wished to conceive [12]. The authors reported significantly higher weight loss following lifestyle intervention compared to usual care. There are few trials in the South Asian population evaluating the role of diet and exercise in PCOS women [16, 17]. One of the smaller trials (*n* = 66) from India reported significant improvement in menstrual symptoms and anthropometric parameters in PCOS women following exercise and metformin [16].

Most of the randomized controlled trials evaluating the role of lifestyle interventions in infertile PCOS women were characterized by small sample size, short follow-up period and high attrition rate [10]. There were no high-quality trials with long-term follow-up evaluating the effectiveness of lifestyle interventions in PCOS women [10]. None of the published trials has included live birth rates as the outcomes, which is one of the important patient-centric fertility endpoints [10]. Furthermore, there is a definite paucity of adequately powered multicentric trials that have examined the efficacy of structured lifestyle intervention in South Asian women with PCOS.

Additionally, the uptake of lifestyle modifications as a treatment strategy in the South Asian population is complicated by a difficult-to-change conventional high-carbohydrate diet and limited availability of space for physical activity in the region [18, 19]. This limits the applicability of findings from some of the earlier trials for the PCOS population from South Asia [11, 12]. For the reasons highlighted above, we plan to conduct an adequately powered multicentric RCT to evaluate the efficacy of individualized lifestyle intervention versus usual care in Indian women with PCOS who wish to conceive.

## Method and design

### Aim of the study

The overall aim of the study is to evaluate the efficacy of individualized lifestyle intervention compared to the usual care in PCOS women who wish to conceive. We hypothesized that individualized lifestyle intervention improves fertility outcomes in PCOS women who wish to conceive when compared to the usual care. To test the hypothesis, we plan to randomize PCOS women into two groups: group A will receive the intervention which will consist of individualized advice on diet and physical exercise along with a telephonic reminder system and follow-up visits, and group B (control) will receive one-time advice on diet and physical exercise. Both groups will receive up to three cycles of ovulation induction with letrozole after 3 months of randomization during the 6-month treatment period.

### Study setting

The study is designed as an open-label, multicentre, superiority, randomized controlled trial. The study has been designed in accordance with the Standard Protocol Items Recommendations for Interventional Trials (SPIRIT) guidelines [20]. The trial has been prospectively registered with the Clinical Trial Registry of India (CTRI/2023/04/051620, registered on 13/4/2023). The sponsor and the funding agency for the trial is the Indian Council of Medical Research (ICMR), New Delhi. The sponsor will be overall responsible for the design, conduct, governance and data management of the trial as well as the final dissemination of the results.

The trial will be conducted at five sites across India which are as follows: (1) Division of Reproductive Medicine, Department of Obstetrics and Gynaecology, All India Institute of Medical Sciences (AIIMS), New Delhi; (2) Department of Reproductive Medicine and Surgery, Christian Medical College (CMC), Vellore, Tamil Nadu; (3) Department of Obstetrics and Gynaecology, Post-graduate Institute of Medical Education and Research (PGIMER), Chandigarh; (4) Department of Obstetrics and Gynaecology, All India Institute of Medical Sciences (AIIMS), Bhubaneswar, Odisha; and (5) Department of Obstetrics and Gynaecology, Sawai Man Singh (SMS) Medical College, Jaipur, Rajasthan. All the sites are medical colleges representing populations from northern, southern, eastern and western regions of India and will have investigators from the endocrine speciality to provide inputs to the design of lifestyle interventions and facilitate the metabolic assessments.

### Inclusion and exclusion

Women attending the fertility clinic will be screened for eligibility. The inclusion criteria will be (i) women aged between 19 and 37 years who have been diagnosed as PCOS by the Rotterdam criteria and wishing to conceive, (ii) body mass index (BMI) ≥ 23 kg/m^2^ but ≤ 35 kg/m^2^ and (iii) duration of infertility ≤ 3 years. The exclusion criteria will be as follows: (i) women planning to take medical treatment at hometown and not available for follow-up period (6 months); (ii) does not have a mobile phone in the house; (iii) pregnancy; (iv) late-onset congenital adrenal hyperplasia (CAH) and androgen secreting tumours; (v) diabetes mellitus, uncontrolled hypothyroidism (TSH > 10 IU/ml), hyperprolactinemia (> 70 ng/ml), bronchial asthma, chronic kidney and liver disease; (vi) prediabetic on metformin or insulin sensitizers or other anti-diabetic medicines; (vii) inflammatory arthritis or any lower limb pathology which precludes exercise; (viii) gastro-intestinal conditions (e.g. ulcerative colitis, Crohn’s disease, coeliac disease) which require special dietary advise; (ix) on anti-psychiatric medications; (x) undiagnosed uterine bleeding; (xi) medical conditions which are contraindications for pregnancy; (xii) previous history of receiving > 6 ovulation induction cycles with letrozole or laparoscopic ovarian drilling; (xiii) previous history of bariatric surgery or planning other strategies for weight loss beyond the trial intervention including but not limited to Fad diets, intra gastric procedures or on weight loss medication; and (xiv) other known causes of infertility which are indications for assisted reproductive technology (ART) treatment, namely severe endometriosis, severe male factor infertility, male partner with azoospermia and documented severe tubal pathology.

Newly detected prediabetics will be included only if they are willing for an initial trial of lifestyle intervention for glycaemic control. We will repeat glycosylated haemoglobin (HbA1c) at 3 months interval, post-randomization, to assess the glycaemic control. If the Hb1Ac is ≥ 6.5%, then initiation of metformin will be considered, and the participant will be followed up as per protocol but will not be considered for per protocol analysis.

The presence of diabetes and prediabetes will be defined based on the criteria from the American Diabetes Association [21]. Individuals with fasting plasma glucose value ≥ 126 mg/dl and/or 2-h plasma glucose value of ≥ 200 mg/dl and/or HbA1c ≥ 6.5% will be diagnosed to have diabetes. Those with a fasting plasma glucose value between 100 and 125 mg/dl with a 2-h plasma glucose < 140 mg/dl (impaired fasting glucose) and/or a 2-h plasma glucose between 140 and 200 mg/dl with a fasting plasma glucose less than 100 mg/dl (impaired glucose tolerance) OR HbA1c (5.7–6.4%) will be diagnosed as pre-diabetics. Those with fasting plasma glucose < 100 mg/dl and a 2-h plasma glucose < 140 mg/dl and HbA1c < 5.7% will be considered to have normal glucose.

### Dropout criteria

Those participants who are randomized but do not report for further follow-up visits at 3 and 6 months will be considered as dropouts. Randomized participants who withdraw their consent also will be considered as dropouts. The participants who report for the follow-up visit but do not comply with dietary and/or exercise advice will be considered as either non-compliant or partially compliant but will not be considered as “dropouts” as per the agreed criteria shown in Table 1.

**Table 1:** Compliance and dropout criteria for inclusion in per-protocol/ITT or additional analysis

**Intervention arm**		
Time point	Diet compliance	Exercise compliance
At 3 months	Point of assessment 1	Point of assessment 2
At 6 months	Point of assessment 3	Point of assessment 4
Ovulation induction with letrozole	Yes	Yes
**Control arm**		
At 6 months	Point of assessment 3	Point of assessment 4
Ovulation induction with letrozole	Yes	Yes

For per protocol analysis inclusion only

• Compliance: for a minimum of three points of assessment and at least one ovulation induction cycle with letrozole (intervention arm)

• Compliance: for both the time points (3 and 4) and at least one ovulation induction cycle with letrozole (for control)

Additional analysis (partial compliance)

• Compliance for at least 2 time points of assessment in the intervention (per protocol will include full compliance and those who were partially compliant) and at least one cycle of ovulation induction

• Compliance at least in 1 time point plus at least one cycle of ovulation induction in the control arm

The following will be included in the intention-to-treat analysis only (not included in per protocol or additional analysis)

• Compliance for less than 2 time points irrespective of ovulation induction for intervention

• Compliance for no time points irrespective of ovulation induction for control

• Did not take ovulation induction irrespective of compliance for both the arms

• Who were dropouts (did not turn up at 6 months)

• Did not receive intervention for at least 4 months

Criteria for deciding non-compliance at each visit:

• Diet: participant consuming > 100 cal over the recommended calories intake

• Exercise: participant GPAQ score < 600

### Ethical issues

All the trial sites have taken ethical clearance from their institutional review board and submitted the same to the sponsor. The trial registration was initiated after receiving the individual ethics approval letters from all the sites and registration was completed on 13.4.23 (CTRI/2023/04/051620). The eligible participants will be invited to participate in the trial, and sufficient time will be given for them to go through trial-related information and make a decision. Those eligible participants who are willing to participate will be recruited for the trial after obtaining written informed consent in their local language. While no ancillary study has been planned in the current trial, we will take an additional consent for obtaining and storing blood samples for future use in an ancillary study.

### Sequence generation and allocation concealment

Once eligibility is confirmed, web-based randomization will be initiated in the presence of the trial staff. The randomization will be done using PHP as the frontend and MySQL as the backend database. Block randomization stratified for the site and BMI (≥ 23–27 kg/m^2^ and > 27 to ≤ 35 kg/m^2^) will be performed. The randomization will be done in a 1:1 ratio to the intervention or the control arm. The trial staff from each site will enter the participant information following which a randomization number will generated and assigned if they fulfil the eligibility criteria. The random number and the allotted arm details will be provided to the trial staff by an authorized person in each site. The allocation will be concealed until the point of randomization. The allotted intervention will be informed to the clinical team as well as the trial staff, and the trial number will be noted.

### Blinding

The nature of the intervention will preclude blinding of the participant and the caregiver. However, the primary and most of the secondary outcomes are objective in nature and are less likely to introduce detection bias. However, performance bias cannot be ruled out.

### Intervention

In the interventional arm, a focused approach to lifestyle interventions involving customized advice on diet and exercise and a follow-up along with medical advice/treatment as per protocol for those seeking fertility treatment will be offered.

For dietary advise, a dietician will assess the usual dietary intake using 24-h dietary recall and food frequency questionnaire (FFQ) at the clinic. The 24-h recall will help to calculate daily macronutrient and micronutrient composition and the quantum of calories consumed per day.

Following a dietary recall, each woman will be given a customized diet plan as per the recent consensus guidelines for the South Asian region [18]. Diet modification will include a reduction in total calories, refined carbohydrates and fats; avoidance of sugar; and inclusion of normal recommended protein and fibre-rich foods. We will monitor the compliance at 3 and 6 months intervals. Compliance will be monitored using a 24-h dietary recall at the fertility clinic.

For the advice on physical activity, a physiotherapist will assess the physical activity using the G-PAQ questionnaire at the fertility/gynaecological clinic. Subjects who are involved in physical labour or who had to walk or cycle for > 30 min/day and were performing exercises regularly will be asked to continue their routine activities. Subjects engaged in sedentary or light physical activity, as assessed in the initial interview will be advised and regularly motivated to walk briskly for at least 30 min each day. These will be customized for each participant based on their physical limitations and current weight. The compliance at 3 and 6 months intervals by using the G-PAQ questionnaire. The follow-up period will be 6 months for each participant.

### Control

Usual care, which is the medical advice/fertility treatment as per unit protocol for those seeking fertility treatment and one time referral to the dietician for dietary advice and exercise by the physiotherapist in the fertility clinic. There will be no planned follow-up for compliance for the lifestyle intervention in the control arm. The control also will receive the fertility treatment after 3 months following randomization if they do not conceive as per unit protocol. The participant will be reviewed after 6 months to measure the anthropometric and reproductive outcomes at the department of reproductive medicine and surgery/gynaecology outpatient clinic. This visit will be combined with any medical treatment-related appointment to avoid multiple visits to the hospital. As mentioned, the participant in the control arm also will receive medical treatment as per unit protocol 3 months post-randomization if she does not conceive.

### Medical treatment 3 months post-randomization for intervention and control arm

These will be as per standard infertility guidelines starting with letrozole. Ovarian induction may be followed with timed intercourse or IUI (combined male factors such as sexual dysfunction) as per the participant’s profile. The treatment will be given for up to 3 cycles depending on the unit policy, while the lifestyle intervention follow-up will continue in the interventional arm over the next 3 months, to be completed for 6 months.

### Dispensing and scheduling of the individualized lifestyle intervention

#### Weeks 0 and 1 (session 1) (for intervention and control arm)

Post-randomization, same day: blood tests (hormonal and metabolic parameters) and anthropometric measurements, including body weight, will be measured. The blood tests will include blood count, serum follicle stimulation hormone (FSH), serum luteinizing hormone (LH), prolactin, thyroid-stimulating hormone (TSH), dehydroepiandrosterone sulphate (DHEAS), 17 hydroxy progesterone, serum testosterone, sex hormone-binding globulin (SHBG), serum insulin, oral glucose tolerance test with 75 g glucose at 0 and 2 h, fasting lipid profile, HbA1c, uric acid and serum adiponectin. The hormonal and other metabolic parameters will be collected at individual sites and sent to a designated central laboratory for analysis. This central accredited laboratory facility will ensure standardization.

A transvaginal ultrasound will be performed and antral follicle counts and ovarian volume (using TVS with a 6.5 to 8.0 MHz probe). The trial staff will then take the anthropometric measurements while the clinician will obtain the ultrasound report. The clinician will also record information on menstrual regularity and pregnancy status. A pregnancy test will be performed if clinically indicated. The trial staff or nurse will record the quality-of-life score using the prespecified tool [22].

**Fig. 1:**
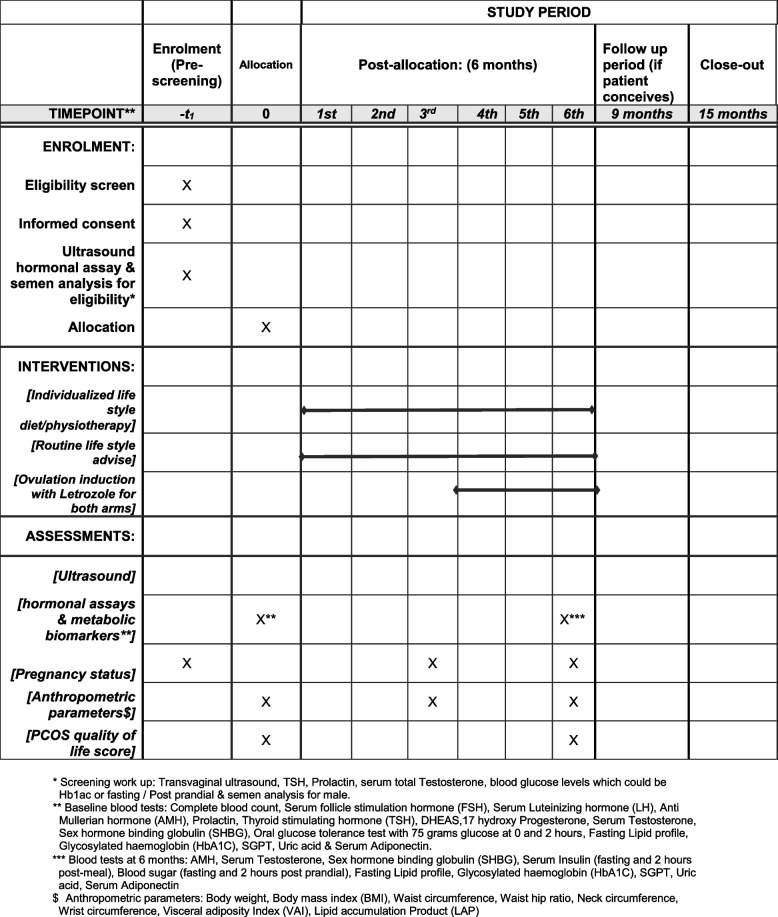
IPOS trial—schedule of enrolment, interventions, and assessments

In-person introduction of the programme and the need for weight loss. Participants will be given the food and activity questionnaire and reviewed the same day/week over the phone/in person (flexible). Based on the questionnaire, the dietician and physiotherapist will advise individualized diet/exercise for the intervention arm. For the control arm, one-time advise on diet and exercise will be given (Fig. 1).

#### Between weeks 2 and 12 (up to the end of the 3rd month) (only for the intervention arm)

Once a week text messages and/or videos on diet and physical exercise to re-in force the intervention.

Once a month telephonic contact to assess compliance with diet/exercise with the participant.

#### Week 13– (session 2) (only for the intervention arm)

In person to check compliance for diet and physical exercise, check weight and anthropometric parameters and as the pregnancy status of the participants. An ultrasound will be performed before initiating ovulation induction.

#### Weeks 14–25 (only for the intervention arm)

Once a week text messages and/or videos on diet and exercise to re-in force the intervention.

Once a month in telephonic contact to assess compliance for diet/exercise with the participant.

#### Week 26– (session 3) (for both the intervention and control arms)

In person check anthropometry and blood tests (hormones and metabolic parameters) and TVS. The following blood tests will be repeated at 6 months: AMH, serum testosterone, SHBG, serum insulin (fasting and 2 h post-meal), blood sugar (fasting and 2 h post-prandial), fasting lipid profile, glycosylated haemoglobin (HbA1C), SGPT, uric acid and serum adiponectin.

Compliance with dietary intake and physical exercise will be assessed for participants in the intervention and control arms using 24 h diet recall and GPAQ tool.

Medical treatment for infertility can be commenced after 12 weeks on post-randomization in case the participant does not conceive. The lifestyle intervention shall stop at 6 months when a final follow-up on anthropometry, metabolic, hormonal and ultrasound will be done in women from both arms. For those who conceive within 6 months post-randomization, the follow-up will extend until delivery/end of pregnancy for recording obstetrics and neonatal outcomes.

### Primary outcome

The primary outcome for the trial will be the live birth following conception during the intervention period (time frame: 0 to 16 months post-randomization for participants who conceive within 6 months following randomization). A live birth is defined as the delivery of a live foetus after 20 weeks of gestation [23]. The twins and higher births will be considered as a single live birth event. The denominator for primary analysis will be per woman.

### Secondary outcomes

There will be several secondary outcomes which will measure clinical, endocrinological and quality of life parameters and are listed below:

(i) Clinical pregnancy rate (time frame: 0 to 9 months post-randomization for participants who conceive within 6 months following randomization): ultrasound evidence of gestational sac or definitive clinical evidence of pregnancy. An ectopic pregnancy will also be considered a clinical pregnancy [24].
(ii) Ongoing pregnancy rate (time frame: 0 to 9 months post-randomization for participants who conceive within 6 months following randomization): a viable intrauterine pregnancy as documented by ultrasound after 12 completed weeks of gestation.
(iii) Miscarriage rate (time frame: 0 to 12 months post-randomization for participants who conceive within 6 months following randomization): miscarriage is defined as a spontaneous loss of pregnancy before 20 completed weeks of gestational age [23].
(iv) Ectopic pregnancy rate (time frame: 0 to 9 months post-randomization for participants who conceive within 6 months following randomization): ectopic pregnancy is defined as pregnancy outside the uterus as diagnosed by ultrasound, surgically or by histopathology [24].
(v) Stillbirth: defined as the death of a foetus prior to the complete expulsion or extraction from its mother after 20 completed weeks of gestational age [23].
(vi) Time to pregnancy (time frame: 0 to 8 months post-randomization for participants who conceive within 6 months following randomization): from randomization to the documentation of pregnancy (calculated in days).
(vii) Positive pregnancy rate (time frame: 0 to 7 months for participants who conceive within 6 months following randomization): defined as positive urine pregnancy test or serum beta human chorionic gonadotrophin (hCG) > 5 IU/ml.
(viii) Mean weight loss (time frame: 6 months post-randomization): difference between baseline and follow-up at 6 months).
(ix) Improvement in menstrual regularity (time frame: 6 months post-randomization): (according to the International Federation of Gynecology and Obstetrics (FIGO) 2018 guidelines variation of the cycle to be called regular is 7–9 days depending on age. Age 18–25 years ≤ 9 days; age 26–41 years ≤ 7 days is regular); proportion of women in whom regularization of the menstrual cycle is recorded by a total number of women in whom menstrual regularity is recorded.
(x) Differences in anthropometric parameters (time frame: 6 months post-randomization) (waist circumference, hip circumference, neck circumference, waist hip ratio, waist height ratio and wrist circumference) at the end of the trial period.
(xi) Improvement in hormonal and metabolic parameters (time frame: 6 months post-randomization) [biochemical markers such as lipid profile and glycaemic parameters, visceral adiposity index (VAI) and lipid accumulation product (LAP)] over the baseline.
(xii) Quality of life (PCOS specific) assessment (time frame: 6 months post-randomization) [22].
(xiii) Pregnancy-related complications (time frame: 0–17 months post-randomization for participants who conceive within 6 months following randomization) (GDM, PIH, preterm birth): obstetric outcomes—gestational diabetes, hypertensive disorder of pregnancy, preterm delivery (< 37 weeks), very preterm birth (< 32 weeks), low birth weight (< 2500 g) or high birth weight (> 4000 g)

### Safety management and adverse events

Any adverse event (AE), which is any untoward medical occurrence, will be documented by the trial site and periodically reviewed by the trial management team. The serious adverse events (SAEs) will be notified to the sponsor, data monitoring safety committee and other sites by the principal investigators (PI) within a stipulated time period of 48 h.

Any adverse event or reaction which (i) is life or limb-threatening, requires hospitalization or a medical intervention or leads to congenital defect is considered a SAE or serious adverse reaction (SAR).

The following will not be included as AE: (i) medical or surgical procedures during the trial (the condition which led to the procedures is deemed an adverse event), (ii) pre-existing condition which did not worsen during trial period and (iii) other unrelated reason for hospitalization (for example, viral fever or incidental illness).

In the intervention arm, if the participant suffers any fracture or other bodily injury which requires hospital care, then it will be considered a notable event. Any food allergy which leads to a hospital visit or admission should be notified. Overall, the lifestyle advise is considered a low-risk intervention and is very unlikely to give rise to any serious adverse events during the trial period.

The participants who conceive during the trial period will be provided with usual ante-natal care based on local unit protocol. Once the woman conceives, the pre-pregnancy lifestyle intervention advised during the trial will be discontinued and pregnancy-specific advice for diet/physical exercise will be given in both arms.

Early pregnancy complications such as threatened, inevitable, incomplete and complete miscarriage and ectopic pregnancy will be recorded. If they are reported during the trial period, they will be recorded as AE or SAE. However, they will not be considered adverse reaction (AR)/serious adverse reaction (SAR) as the intervention is unlikely to have a causal relationship with the early pregnancy complications. Obstetric complications, namely, pregnancy-induced hypertension, pre-eclampsia and gestational diabetes, will be recorded during the follow-up period. The low birth weight and pre-term birth also will be recorded. Since these outcomes will be observed after the intervention period, they will not be recorded as adverse events.

Medical procedures/surgical procedures performed to manage pregnancy complications will be recorded as well. They will not be recorded as AE, but the reason for the medical/surgical procedure will be recorded as AE. The trial site staff will inform the PI/Co-PI within 48 h of recording the SAE and enter the same in the database. If the event requires activation of referral or further hospital care, the process should be facilitated. For other AEs, the trial staff should enter the event in the database within 7 days of recording the event.

### Data collection and management

The data related to clinical characteristics, anthropometric measurements and endocrinological parameters of the participants at the baseline will be collected. Subsequent information collected at the follow-up along with outcome data will be captured at the end of the treatment period. The data will be collected from the source data and entered into the central database by the data operator. Each entry will be double-checked by one of the trial staff. Finally, as a quality control measure, 10% of the entries will be validated by the central monitoring team who will cross-verify the entries by comparing the CRF/source data, if deemed necessary. The CRFs provided to the central monitoring team will carry only anonymised participant-related data. The data access will be restricted to designated trial staff at each site and the members of the sponsoring agency, ICMR to maintain data confidentiality.

### Data monitoring

The central data monitoring committee will be set up and meet before the trial starts recruitment. The group will have access to confidential information as well as accumulating data including SAEs. The data monitoring committee (DMC) will review the trial data routinely and using statistical analysis, inform the trial steering committee about the following: (i) unequivocal evidence that one arm is doing poorly compared to the other arm as mentioned under stop trial conditions, (ii) good evidence that one arm better than the other as mentioned under stop trial conditions, (iii) any protocol changes and (iv) safety data review. However, we do not anticipate any major safety concerns due to the nature of the intervention.

The DMC will consist of trial statistician as well as independent members who are not part of the trial and submit periodic reports to the trial steering committee (TSC). All the members will be treatment blind except the statistician. They will also conduct the interim analysis at 30 months.

### Trial oversight

We will constitute a trial managing group (TMG) consisting of PIs/co-PIs and trial managers from all the sites, the trial statistician and representatives from the sponsor. The TMG will meet at prespecified intervals for reviewing and coordinating the conduct of the trial at the site level. The TSC will also be formed for the trial governance. The TSC will have members from the TMG (PIs and trial statistician), independent members and Patient and Public Involvement (PPI) representative. The main role of TSC will be to provide overall supervision for the trial and give advice through its independent chair on trial-related matters. The final decision regarding the continuation of the trial will rest with the TSC. The TSC will also oversee the dissemination of the results of the trial and the publication process.

### Recruitment strategies

All the sites are large tertiary care centres with a high volume of clinical work and eligible population. We do not anticipate any difficulty in recruitment. However, we will monitor the recruitment rate per site every 6 months. During the monthly TMG meetings, those sites with slower accrual (less than five participants per site per month) will be advised to identify the reasons for the lower recruitment rate and take the necessary corrective steps. Furthermore, we have a review plan to review site performance after 1 year and in case of any slow accrual from any site, we will allocate additional participants to other performing sites.

### Patient and public involvement

Members from the public as well the patients will be invited, and a group will be formed which will help at the design stage as well as be involved in trial governance through TSC.

### Ancillary study

The ancillary study has not been planned for the current project. However, the individual sites can explore translational research at individual sites after seeking permission from TSC as well as seek fresh ethics committee approval and separate funding for the same.

Additional blood samples will be stored for possible future translational research. Written informed consent incorporates the same and the participants will be informed about the future study.

### Sample size calculation

No previous data is available on live birth following individualized lifestyle intervention in PCOS women who wish to conceive. From previous study performed in one of the trial sites, the live birth rate following usual care which consisted of one-time lifestyle advise and ovulation induction with Letrozole for up to five treatment cycles, was reported as 30% [25]. Due to uncertainty about the likely event rate (live birth rate) in the intervention arm, it was assumed that to convince the clinicians and patients to adopt a change in practice, a modest improvement of 10% in live birth would be required.

For alpha error set at 0.05 and power being 90%, to detect a clinically relevant difference of 10% in the live birth rate following individualized lifestyle intervention, the calculated sample size will be 473 in each arm (total sample size 946). Assuming an attrition of 20%, the total sample size was 1183. We rounded off the total sample size to 1200 with 600 in each arm. With 5 centres participating in the study each site shall recruit 240 subjects each.

### Statical analysis

Data will be entered using ICMR software (developed by ICMR, New Delhi) and screened for outliers and extreme values using Box-Cox plot and histogram (for the shape of the distribution). Summary statistics will be used for reporting demographic and clinical characteristics using mean (SD)/median (range) for the continuous variables, and frequency along with percentage for categorical variables. An intention-to-treat analysis and per-protocol analysis for the primary and secondary endpoints will be performed. The continuous endpoints will be tested using *z*-test for means, and the difference in means will be presented with 95% CI. The categorical endpoints will be tested using *z*-test for proportions and the difference in proportion will be presented with 95% CI. A generalized estimating equations (GEE) will be performed to understand the compliance and the effect over time between the two groups of intervention. To understand the advantage of intervention the number needed to treat will be presented.

Based on BMI 23–27 and > 27–35 kg/m^2^, we will perform subgroup analysis to explore whether there is any difference in the effect of the intervention in different subpopulations.

All the randomized subjects will be included in the intention to-treat (ITT) analysis. The ITT analysis is done for the primary endpoint and secondary endpoints and analysed according to the group assigned by randomization. For dropouts with no information on the outcome, it will be assumed that the intervention did not result in a live birth (Table 1).

Per-protocol set (PPS) is the subset of ITT, where the subjects are randomized and treated without any major protocol deviations that impacts the efficacy of the results. The PSS will be used for primary endpoint analysis and secondary end-points. The analysis set will be the subset of participants who are not dropped out of the study and satisfy the protocol requirement. Non-compliant will not be considered for the per-protocol analysis but will be included in the ITT (Table 1). We will perform additional analysis for partially compliant participants and include them in the per-protocol analysis (Table 1). The analysis will be performed by creating an interaction term for the intervention group with partially and fully complaint groups presenting multiple testing-adjusted *p*-values.

We plan to adjust the results for the following covariates: (i) number of letrozole cycles. Logistic regression analysis will be used to adjust the covariates effectively the adjusted effect size will be presented as an odds ratio (95% CI) for binary outcomes. All the analyses will be performed using STATA/IC 16.0.

### Protocol amendments

The final protocol was approved on 2 April 2023. During the conduct of the trial, if any major protocol amendment is made, we plan to inform the ethics committee of all the sites, the sponsor as well as the TSC.

## Discussion

Lifestyle intervention is considered as the first line of management for treating PCOS women who present with infertility. While earlier studies have shown some improvement in menstrual symptoms, endocrinological parameters and ovulation rate, very few have reported clinically important fertility outcomes, i.e. live birth [11–14]. The current trial is planned to capture all the relevant clinical domains which are important to the PCOS women such as reproductive outcomes, improvement in menstrual symptoms, changes in anthropometric measurements and endocrinological parameters and, finally, the quality-of-life score.

High dropout rates and adherence issues are some of the challenges in trials evaluating lifestyle intervention [10, 26]. The Cochrane review evaluating the lifestyle intervention reported high dropout rates (up to 46%), especially in studies involving longer treatment periods [10]. A systematic review investigated the dropout rates in studies evaluating the lifestyle intervention in overweight and obese infertile women [26]. The review included 15 studies, out of which 10 reported the dropout rate. The median dropout rate was 24% (range 0–31%). The authors could not find any modifiable predictors for dropouts and reported lower weight loss in women who had dropped out of the trial. The current study has calculated the sample size taking into account the possibility of high attrition (20%) that may be encountered during the conduct of the trial. In the currently planned trial, it has been hypothesized that customized lifestyle intervention delivered by the medical staff other than the treating clinician may help in greater adherence and lower dropouts. The study will indirectly help us investigate the impact of such an approach on the attrition rate for the lifestyle intervention.

One of the reasons for planning the current study is the limited applicability of trial findings conducted in other parts of the world, such as Europe and North America due to marked differences in dietary patterns [27]. Furthermore, there are also differences in the level and nature of daily physical activity performed by women from South Asian region versus women from European or North America [28]. Some of the urban areas in South Asia have problems with limited public space and access to sporting facilities. Hence, it is important to customize the lifestyle interventions depending on each individual’s preferences and availability of resources. Moreover, the South Asian phenotype related to obesity is prone to developing complications at a lower body mass index and more resistant to lifestyle intervention [29, 30].

An adequately powered sample size is one of the main strengths of the current study. Involving multiple sites across the different regions of the country will help in the generalizability of the findings. It is hypothesized that the customized lifestyle intervention for PCOS women primarily involving the dietician and physiotherapist and reducing the involvement of medical doctors may help improve compliance. In low-resource settings, the medical doctor is often overburdened and may not be able to deliver such time-consuming interventions in an optimum manner. The intervention, if found to be beneficial, can be replicated in real-world clinical practice, especially in a low-resource setting. One of the limitations of the study is the difficulty in assessing adherence to the given dietary and exercise-related advise. While it is possible to add additional monitoring tools such as electronic gadgets for exercise and measuring diet specific marker or metabolite in blood or urinary sample, we avoided using such tools as it is difficult to replicate such measures in real-world practice, especially in low-resource setting.

Through this protocol, we would like to connect with other investigators who are planning to initiate similar trials or have ongoing projects investigating lifestyle interventions in women with PCOS. We would like to invite the investigators of such projects to join us through a collaborative framework such as prospective meta-analysis or data sharing arrangement plan for future individualized participant metanalysis [31].

The guidelines have always emphasized the lifestyle intervention as the first line of management for PCOS women [9]. However, these recommendations are mostly based on data from smaller trials with limited generalizability. The current trial results could help clarify and provide more robust evidence for advocating an individualized lifestyle intervention in PCOS women who wish to conceive.

## Trial status

The trial will be open for recruitment from 1 May 2023. We expect to complete the recruitment of all the participants by March 2027 and the follow-up by December 2027. The protocol version 1 was approved on 2 April 2023.

## Supplementary Information

**Additional file 1.**

## Data Availability

The final IPOS trial dataset will be stored and maintained at the ICMR, New Delhi. The data request proposal will be overseen by the IPOS steering committee along with ICMR, and their decision will be final and binding. For those data requests which are permitted, the data transfer will need to comply with the data transfer agreement guidelines.

## References

[1] The Rotterdam ESHRE/ASRM-sponsored PCOS consensus workshop group (2004). Revised 2003 consensus on diagnostic criteria and long-term health risks related to polycystic ovary syndrome (PCOS). Hum Reprod.

[2] Liu J, Wu Q, Hao Y, Jiao M, Wang X, Jiang S (2021). Measuring the global disease burden of polycystic ovary syndrome in 194 countries: Global Burden of Disease Study 2017. Hum Reprod.

[3] Mani H, Davies MJ, Bodicoat DH, Levy MJ, Gray LJ, Howlett TA (2015). Clinical characteristics of polycystic ovary syndrome: investigating differences in White and South Asian women. Clin Endocrinol (Oxf).

[4] Kim JJ, Choi YM (2019). Phenotype and genotype of polycystic ovary syndrome in Asia: ethnic differences. J Obstet Gynaecol Res.

[5] Azziz R, Carmina E, Dewailly D, Diamanti-Kandarakis E, Escobar-Morreale HF, Futterweit W (2009). The Androgen Excess and PCOS Society criteria for the polycystic ovary syndrome: the complete task force report. Fertil Steril.

[6] ESHRE Capri Workshop Group (2012). Health and fertility in World Health Organization group 2 anovulatory women. Hum Reprod Update..

[7] Hart R, Hickey M, Franks S (2004). Definitions, prevalence and symptoms of polycystic ovaries and polycystic ovary syndrome. Best Pract Res Clin Obstet Gynaecol.

[8] Franks S, Stark J, Hardy K (2008). Follicle dynamics and anovulation in polycystic ovary syndrome. Hum Reprod Update.

[9] Teede HJ, Misso ML, Costello MF, Dokras A, Laven J, Moran L (2018). Recommendations from the international evidence-based guideline for the assessment and management of polycystic ovary syndrome^†‡^. Hum Reprod.

[10] Lim SS, Hutchison SK, Van Ryswyk E, Norman RJ, Teede HJ, Moran LJ (2019). Lifestyle changes in women with polycystic ovary syndrome. Cochrane Database Syst Rev.

[11] Legro RS, Dodson WC, Kris-Etherton PM, Kunselman AR, Stetter CM, Williams NI (2015). Randomized controlled trial of preconception interventions in infertile women with polycystic ovary syndrome. J Clin Endocrinol Metab.

[12] Jiskoot G, Timman R, Beerthuizen A, Dietz de Loos A, Busschbach J, Laven J. Weight reduction through a cognitive behavioral therapy lifestyle intervention in PCOS: the primary outcome of a randomized controlled trial. Obesity. 2020;28(11):2134–41. 10.1002/oby.22980 PMC7702123 32969197

[13] Thomson RL, Buckley JD, Noakes M, Clifton PM, Norman RJ, Brinkworth GD (2008). The effect of a hypocaloric diet with and without exercise training on body composition, cardiometabolic risk profile, and reproductive function in overweight and obese women with polycystic ovary syndrome. J Clin Endocrinol Metab.

[14] Palomba S, Giallauria F, Falbo A, Russo T, Oppedisano R, Tolino A (2008). Structured exercise training programme versus hypocaloric hyperproteic diet in obese polycystic ovary syndrome patients with anovulatory infertility: a 24-week pilot study. Hum Reprod.

[15] Benham JL, Booth JE, Corenblum B, Doucette S, Friedenreich CM, Rabi DM (2021). Exercise training and reproductive outcomes in women with polycystic ovary syndrome: a pilot randomized controlled trial. Clin Endocrinol (Oxf).

[16] Tiwari N, Pasrija S, Jain S (2019). Randomised controlled trial to study the efficacy of exercise with and without metformin on women with polycystic ovary syndrome. Eur J Obstet Gynecol Reprod Biol.

[17] Saraswat S. Vegan or low calorie diet for weight loss in polycystic ovary syndrome females: a randomised controlled study. Stud ETHNO-Med. 2020;14(1–2). [cited 2023 Feb 23]: Available from: http://krepublishers.com/02-Journals/S-EM/EM-14-0-000-20-Web/S-EM-14-0-000-20-Contents/EM-14-0-000-20-Contents.htm.

[18] Kapoor N, Sahay R, Kalra S, Bajaj S, Dasgupta A, Shrestha D (2021). Consensus on Medical Nutrition Therapy for Diabesity (CoMeND) in adults: a South Asian perspective. Diabetes Metab Syndr Obes Targets Ther.

[19] Yiga P, Ogwok P, Achieng J, Auma MD, Seghers J, Matthys C (2021). Determinants of dietary and physical activity behaviours among women of reproductive age in urban Uganda, a qualitative study. Public Health Nutr.

[20] Chan AW, Tetzlaff JM, Altman DG, Laupacis A, Gøtzsche PC, Krleža-Jerić K (2013). SPIRIT 2013 statement: defining standard protocol items for clinical trials. Ann Intern Med.

[21] ElSayed NA, Aleppo G, Aroda VR, Bannuru RR, Brown FM, Bruemmer D, et al. on behalf of the American Diabetes Association. 2. Classification and diagnosis of diabetes: standards of care in diabetes-2023. Diabetes Care. 2023;46(Suppl 1):S19-S40. 10.2337/dc23-S002 PMC9810477 36507649

[22] Williams S, Sheffield D, Knibb RC (2018). The Polycystic Ovary Syndrome Quality of Life scale (PCOSQOL): development and preliminary validation. Health Psychol Open.

[23] Duffy JMN, Bhattacharya S, Bhattacharya S, Bofill M, Collura B, Curtis C (2021). Standardizing definitions and reporting guidelines for the infertility core outcome set: an international consensus development study. Fertil Steril.

[24] Zegers-Hochschild F, Adamson GD, Dyer S, Racowsky C, de Mouzon J, Sokol R (2017). The international glossary on infertility and fertility care, 2017. Fertil Steril.

[25] Waanbah BD, Joseph T, Rebekah G, Kunjummen AT, Kamath MS (2021). Letrozole as first-line drug for ovulation induction in treatment-naïve infertile polycystic ovarian syndrome women. J Obstet Gynaecol Res.

[26] Mutsaerts MAQ, Kuchenbecker WKH, Mol BW, Land JA, Hoek A (2013). Dropout is a problem in lifestyle intervention programs for overweight and obese infertile women: a systematic review. Hum Reprod.

[27] Simmons D, Williams R (1997). Dietary practices among Europeans and different South Asian groups in Coventry. Br J Nutr.

[28] Sriskantharajah J, Kai J (2006). Promoting physical activity among South Asian women with coronary heart disease and diabetes: what might help?. Fam Pract.

[29] Kapoor N, Lotfaliany M, Sathish T, Thankappan KR, Thomas N, Furler J (2020). Prevalence of normal weight obesity and its associated cardio-metabolic risk factors - results from the baseline data of the Kerala Diabetes Prevention Program (KDPP). PLoS ONE.

[30] Kapoor N, Lotfaliany M, Sathish T, Thankappan KR, Tapp RJ, Thomas N (2020). Effect of a peer-led lifestyle intervention on individuals with normal weight obesity: insights from the Kerala Diabetes Prevention Program. Clin Ther.

[31] Seidler AL, Hunter KE, Cheyne S, Ghersi D, Berlin JA, Askie L (2019). A guide to prospective meta-analysis. BMJ.

