# Publisher Correction: Individualized lifestyle intervention in PCOS women (IPOS): a study protocol for a multicentric randomized controlled trial for evaluating the effectiveness of an individualized lifestyle intervention in PCOS women who wish to conceive

**DOI:** 10.1186/s13063-023-07535-2

**Published:** 2023-07-31

**Authors:** Neena Malhotra, Taruna Arora, Vanita Suri, Saubhagya Kumar Jena, Asha Verma, Mahasampath Gowri, Nitin Kapoor, Manjeet Singh Chalga, Bharati Kulkarni, Mohan S. Kamath

**Affiliations:** 1 Division of Reproductive Medicine, Department of Obstetrics and Gynaecology, All India Institute of Medical Sciences, New Delhi, India; 2 Division of Reproductive and Child Health and Nutrition, Indian Council of Medical Research, New Delhi, India; 3 Department of Obstetrics and Gynaecology, Post-Graduate Institute of Medical Education and Research, Chandigarh, India; 4 Department of Obstetrics and Gynaecology, All India Institute of Medical Sciences, Bhubaneswar, Odisha India; 5 Department of Obstetrics and Gynaecology, Sawai Man Singh Medical College, Jaipur, Rajasthan India; 6 Department of Biostatistics, Christian Medical College, Vellore, Tamil Nadu India; 7 Department of Endocrinology, Diabetes and Metabolism, Christian Medical College, Vellore, India; 8 Non-Communicable Disease Unit, Baker Heart and Diabetes Institute, Melbourne, VIC Australia; 9 Division of Biomedical Informatics, Indian Council of Medical Research, New Delhi, India; 10 Department of Reproductive Medicine and Surgery, Christian Medical College, Vellore, Tamil Nadu India

**Publisher Correction: BMC Trials 24, 457 (2023)**

**https://doi.org/10.1186/s13063-023-07466-y**

Following publication of the original article [1], we have been made aware that one of the co-authors’ names was incorrectly edited during proofing to Taruna Katyal Arora instead of Taruna Arora.

The original article has been corrected.
