# Supplementary material for: Individualized lifestyle intervention in PCOS women (IPOS): a study protocol for a multicentric randomized controlled trial for evaluating the effectiveness of an individualized lifestyle intervention in PCOS women who wish to conceive
Source: Trials. 2023 Jul 18;24:457. doi: 10.1186/s13063-023-07466-y (PMC10353229; doi:10.1186/s13063-023-07466-y)
Supplement: Supplementary file 1 — Additional file 1. [file 13063_2023_7466_MOESM1_ESM.docx]

**Department of Reproductive Medicine & Surgery, Christian Medical College, Vellore- 632004**

Title of the study: Effectiveness of an individualized lifestyle intervention in PCOS women who wish to conceive: a multi-centered randomized controlled trial.

**Information sheet**

Polycystic ovarian syndrome (PCOS) is a common health condition affecting 10% of the women in the age group of 15-40 years and it is an important cause of infertility. PCOS plays an important role in the reproductive and metabolic function. The women with PCOS are diagnosed based on medical criteria, if they satisfy two of the three criteria i.e., 1) infrequent menses or no menses, 2) excess male hormone production in the body which may be associated with excessive body hair growth such as facial hair, 3) presence of polycystic ovaries on ultrasound. Most of the women with PCOS have associated obesity and reduced insulin function. PCOS may lead to long term complications like diabetes, high blood pressure, heart blood vessel related issues, if adequate care is not taken. In PCOS women, the infrequent menses indicate a delayed release or no release of egg which may lead to decreased chances of natural conception.

Lifestyle interventions are recommended as first line treatment in PCOS. These include mainly modification in the diet and exercise. These interventions are beneficial in the overweight or obese PCOS women and help in improving the menstrual irregularities and the metabolic functions. But the main problem associated is the inability to follow the advice given by the doctor due to family or work-related issues. We are conducting a study to check the feasibility of lifestyle modifications which have been advised by the doctor, in PCOS women seeking fertility treatment. This study would help us understand if you were able to follow the advice correctly or whether there were any obstacles to the follow up and the reasons for not being able to follow up. We would also be checking if the diet modification and exercise has helped you in reducing your weight and achieving a pregnancy. Participation in this study will not affect your fertility treatment.

**If you take part, what will you have to do?**

The main aim of our study is to see the investigate of lifestyle intervention in PCOS women who desire fertility*.*  You shall be invited to participate in our study. There are two groups in our study. If you agree to participate, you will be assigned to either one of two study groups. This will be decided by a computer program; this is like tossing a coin and you have an equal chance of getting assigned in either group. If you are assigned to the intervention group, you will be assessed by a reproductive medicine doctor initially then you will get referred to dietician/physiotherapist for targeted lifestyle intervention which includes diet and exercise management, and you will be evaluated once after 3 months, and second time at 6 months regarding your progress and adherence to the medical advice. If you are assigned to the control group, you will be provided one time diet & exercise advise and you will be evaluated after 6 months regarding your progress and adherence to the medical advice. All other treatments that you are already on will be continued and your regular fertility treatment will not be changed during this study in both the groups. Additional blood tests will be conducted once at the time of study and at the end of the study in both the groups but you will not be charged for these blood tests.

**Can you withdraw from this study after it starts?**

Your participation in this study is entirely voluntary and you are also free to decide to withdraw permission to participate in this study. If you do so, this will not affect your usual treatment at this hospital in any way. In addition, if you experience any serious side effects or your condition worsens, the study will be stopped, and you may be given additional treatment.

**Are there any risks involved by participating in the study?**

We do not expect any risks but if you do develop any problems due to the study, these will be assessed and treated.

**What happens after the study is over?**

You may or may not benefit directly from the study. Once the study is over if the intervention is found effective, we may use it for other patients, undergoing the same procedure

**Will your personal details be kept confidential?**

The results of this study will be published in a medical journal, but you will not be identified by name in any publication or presentation of results. However, your medical notes may be reviewed by people associated with the study within our hospital and ICMR, New Delhi, without your additional permission, should you decide to participate in this study.

**If you have any queries, you can contact us**

Dr Mohan S Kamath (principal investigator)

Professor and Head,

Department of Reproductive Medicine and Surgery

CMC Vellore., phone: 04162283302; cell number 9994497690; Email: [mohankamath@cmcvellore.ac.in](mailto:mohankamath@cmcvellore.ac.in)**.**

**Date:**

**Informed Consent form to participate in a research study**

**Study Title: Effectiveness of an individualized lifestyle intervention in PCOS women who wish to conceive: a multi-centered randomized controlled trial.**

**Study Number: ____________**

**Subject’s Initials: __________________ Subject’s Name: _________________________________________**

**Date of Birth / Age: ___________________________**

(Subject)

(i) I confirm that I have read and understood the information sheet dated ____________ for the above study and have had the opportunity to ask questions. [ ]

(ii) I understand that my participation in the study is voluntary and that I am free to withdraw at any time, without giving any reason, without my medical care or legal rights being affected. [ ]

(iii) I understand that, the Ethics Committee, the regulatory authorities and Indian Council of Medical Research, New Delhi will not need my permission to look at my health records both in respect of the current study and any further research that may be conducted in relation to it, even if I withdraw from the trial. I agree to this access. However, I understand that my identity will not be revealed in any information released to third parties or published. [ ]

(iv) I agree not to restrict the use of any data or results by the trial investigators and Indian Council of Medical Research, New Delhi that arise from this study provided such a use is only for scientific purpose(s). [ ]

(iv) I agree to give blood samples which will be analysed for the current study purpose as well as for future studies. [ ]

(v) I agree to take part in the above study. [ ]

Signature (or Thumb impression) of the Subject/Legally Acceptable

Date: _____/_____/______

Signatory’s Name: _________________________________ Signature:

Or

Signature of the Investigator: ________________________

Date: _____/_____/______

Study Investigator’s Name: _________________________

Signature or thumb impression of the Witness: ___________________________

Date: _____/_____/_______

Name & Address of the Witness: ______________________________
